# Influence of Molecular Mobility on Contrast Efficiency of Branched Polyethylene Glycol Contrast Agent

**DOI:** 10.1155/2018/1259325

**Published:** 2018-12-02

**Authors:** Yu-I Hsu, Atsushi Mahara, Tetsuji Yamaoka

**Affiliations:** Department of Biomedical Engineering, National Cerebral and Cardiovascular Center Research Institute, 5-7-1 Fujishiro-dai, Suita, Osaka 565-8565, Japan

## Abstract

For a water-soluble polyethylene glycol (PEG) magnetic resonance imaging (MRI) contrast agent, it has been demonstrated that the contrast efficiency was increased with increased branched structure of the contrast agent. However, the cause of enhanced contrast efficiency by the branched structure has not been clarified. Hence, we investigate the cause of the contrast agent enhancement by changing the Gd introduction ratio of the eight-arm PEG from 1.97 to 4.07; furthermore, the terminal mobility of the contrast agents with different structures was evaluated using proton nuclear magnetic resonance (^1^H-NMR) spectroscopy. It was shown that the relaxivity and contrast luminance of the synthesized branched PEG-Gd contrast agents are larger than those of linear PEG-Gd and commercially available contrast agents. Additionally, the change in the Gd introduction ratio did not affect the contrast efficiency. The terminal mobility results measured by NMR show that the linewidth at half height became broader with an increased number of branches, implying that the mobility of branched PEG-Gd is slower than that of linear PEG-Gd. Interestingly, the linewidth at half height of different structures did not change in an organic solvent; this phenomenon appeared specifically in water. It is suggested that the stable branched structure enabled the improvement in the relaxivity and contrast luminance.

## 1. Introduction

Recently, effective Gd-based MRI contrast agents for magnetic resonance imaging (MRI) and magnetic resonance angiography (MRA) have attracted particular attention [1–6]. Using a water-soluble polymer, the contrast agents leaked out easily from the injection site and were excreted from the body, thereby reducing side effects in a patient. In our previous study, we used polyvinyl alcohol (PVA) or dextrane (Dex) as the primary chains to develop A novel water-soluble MRI contrast agent for in vivo living cell tracking [7–10]. After cell labeling using PVA-Gd and Dex-Gd, the signal enhancement of cells was observed in vitro and in vivo, and the contrast agent leaked out from dead cells and was excreted from the body when the transplanted cell died. Moreover, the effect of PVA-Gd labeling on mesenchymal stem cell (MSC) proliferation was much less than that of the commercially available contrast agent ProHance, and the labeled MSCs were found to have osteoblastic differentiation ability [11]. Because the molecular weight and the injection site are the important factors that affect the concentration profile of polymers in the blood circulation [12], the use of poly(ethylene glycol) (PEG) with lower molecular weight to synthesize the contrast agent is necessary. Unlike PVA and Dex, which have multiple functional groups that can react with Gd, PEG can react only with two functional groups at the terminal; therefore, using branch-structured PEG can increase the reactive functional groups to increase the Gd chelate in one polymer.

Some reports indicated that dendrimers conjugated with Gd chelates and magnetic tags can enhance the relaxivity of the contrast agents [13–18]. Klemm et al. reported that biocompatible macromolecular dendrimer conjugates have high relaxivity and bioavailability and low in vitro toxicity. Margerum et al. investigated the relationship between relaxivity and dendrimer generation, where high generation (G5) was revealed with a higher relaxivity of up to 18.8 mM^−1^·s^−1^. It was demonstrated that the relaxivity of a dendrimer's Gd chelate is related to its mobility and that a high relaxivity rate is generally induced by a low mobility. High relaxivity can also be achieved by conjugation with macromolecules such as virus capsids [19, 20], nanoparticles [21], and liposomes [22, 23]. Manus et al. prepared a Gd(III)-nanodiamond conjugate [Gd(III)-ND] with three Gd-chelate molecules. The Gd(III)-ND particles significantly reduced the T1 of the water protons with a per-Gd(III) relaxivity of 58.82 ± 1.18 mM^−1^·s^−1^ at 1.5 T (60 MHz), and its relaxivity exhibited a 10-fold increase compared to the monomer Gd(III) complex (*r*1 = 5.42 ± 0.20 mM^−1^·s^−1^). Because PEG could reduce the toxicity and the reticuloendothelial system recognition of the nanoparticles, some researches are using PEG as the modifier [24–26]. Recently, we reported an MRI contrast agent using branched PEG as the main chain [27]. A branched eight-arm PEG-Gd having two or three Gd chelate molecules showed higher relaxivity than linear PEG-Gd and commercially available contrast agents, e.g., ProHance. The molecular weight of PEG is smaller than that of macromolecules such as dendrimers, liposomes, and nanodiamond conjugates; however, the cause of the enhanced contrast efficiency by the branch-structured PEG has not been clarified. Jiang et al. used variable-temperature NMR measurements to study the phase transitions of novel glycolides with pendent oligo(ethylene oxide)monomethyl ether substituents [28]. They found that when heated to 25°C, the peak broadened and the peak height noticeably decreased, indicating a phase transition from a soluble to an insoluble state between approximately 18°C and 25°C, consistent with the cloud point measurements. When the peak reached the cloud point, the molecular mobility became lower, the relaxation time of water became shorter, and the peak became broader.

In this study, we synthesized linear PEG-Gd, dendron PEG-Gd, four-arm PEG-Gd, and eight-arm PEG-Gd; moreover, eight-arm PEG-Gds having different Gd-chelate molecule ratios from two to four were also synthesized. The longitudinal relaxation time (*T*1, ms) and transverse relaxation time (*T*2, ms) of the synthesized PEG contrast agent solutions were characterized by NMR measurements, and the *T*1-weight images were obtained by 1.5 T MRI. The contrast efficiency, zeta potential, and hydrodynamic radius of the synthesized PEG contrast agents were compared with commercially available contrast agents, Magnescope and ProHance. These synthesized PEG-Gds were also evaluated by the linewidth at half height of each PEG contrast agent through NMR measurements to investigate the cause of the contrast agent enhancement. The NMR measurements were performed at different temperatures and with deuterated solvents. From these measurements, we successfully clarified the relationship between the structure and the contrast efficiency of the PEG contrast agents.

## 2. Materials and Methods

### 2.1. Materials

The PEG derivatives, hexaglycerol octa(aminopropyl) polyoxyethylene (eight-arm PEG-NH_2_, Mn = 15000), pentaerythritol tetra(aminopropyl) polyoxyethylene (four-arm PEG-NH_2_, Mn = 15000), 2,3-bis-[2′,3′-di(methylpolyoxyethylene-oxy)-1′-propyl]polyoxyethylene-oxy-1-(aminopropyloxy) propane (dendron PEG-NH_2_, Mn = 40000), and *α*-aminopropyl-*ω*-aminopropoxy polyoxyethylene (linear PEG-NH_2_, Mn = 10000) were purchased from NOF CORPORATION (Tokyo, Japan). 1,4,7,10-Tetraazacyclododecane-1,4,7,10-tetraacetic acid mono(*N*-hydroxysuccinimidyl ester) (DOTA-NHS-ester) was purchased from NARD Institute Ltd. (Hyogo, Japan). Gadolinium chloride (GdCl_3_) was purchased from Wako Pure Chemical Industries (Osaka, Japan). Other reagents and solvents were commercially available and used as received.

### 2.2. Measurements

The elemental composition was determined using the ICPM-8500 inductively coupled plasma mass spectrometer (Shimadzu Co., Japan). The hydrodynamic radii and zeta potential were determined by a Malvern zeta nanosizer (Malvern Instruments Ltd., UK). The MR images were determined by the MRmini small animal MRI (Pharma Biomedical Co., Japan) operating at 1.5 T. The proton nuclear magnetic resonance (^1^H-NMR) spectroscopy was conducted using a Gemini-300 NMR spectrometer (Varian Inc., USA) at 300 MHz (7.1 T).

### 2.3. Synthesis of Contrast Agents

**Scheme 1:**
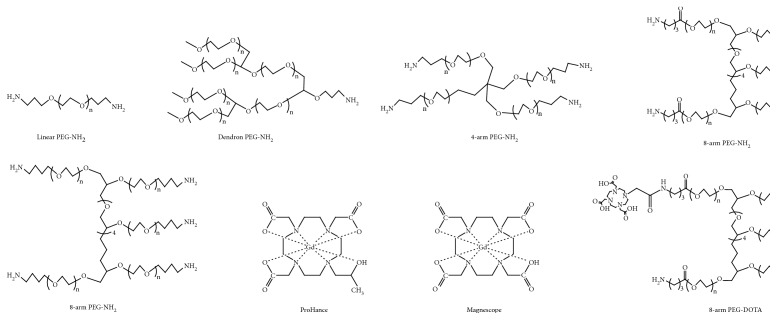
(a) Chemical structure of linear, dendron, four-arm, and eight-arm PEG-Gd. (b) Typical two-step synthesis of PEG-Gd.

Scheme 1 shows the structure and the synthesis of the PEG contrast agents of different structures. The PEG contrast agents were synthesized by referring our previous report [27]. Briefly, the PEG derivatives, eight-arm PEG-NH_2_, four-arm PEG-NH_2_, dendron PEG-NH_2_, and linear PEG-NH_2_ (Scheme 1(a)) were reacted with DOTA-NHS-ester (three-mole equivalent to amino acid terminal of PEG-NH_2_) in anhydrous dimethyl sulfoxide (DMSO) under nitrogen atmosphere at room temperature for one day. After the reaction, the products were purified by membrane dialysis using a spectra/pore membrane (MWCO = 3500) in distilled water, thrice. The purified solution was subsequently freeze-dried to obtain PEG-DOTA (Scheme 1(b) Step 1). The obtained PEG-DOTA was subsequently dissolved in distilled water and reacted with the dropwise addition of 1.5 mole equivalent of GdCl_3_ to the DOTA while stirring. The pH was maintained between 6.5 and 7.0 with 0.1 M NaOH solution and stirred overnight at room temperature. The reacted mixture was subsequently dialyzed in distilled water thrice and freeze-dried to obtain PEG-Gd (Scheme 1(b) Step 2). The ratios of the DOTA terminal and Gd conjugation were measured by ^1^H-NMR and ICP-MS, respectively. The hydrodynamic radius and zeta potential were determined by the Malvern zeta nanosizer.

### 2.4. Measurements of Relaxivity and *T*1-Weighted MR Images

The longitudinal relaxation time (*T*1, ms) and transverse relaxation time (*T*2, ms) of the synthesized PEG contrast agent solutions were measured using a 300 MHz (7.1 T) NMR spectrometer with a combination of measurements obtained in a large NMR tube (650 *μ*L of deuterium oxide to dilute the synthesized PEG contrast agents to different concentrations from 0.05 mM to 0.8 mM) and in a small tube (containing 50 *μ*L of benzene-D6). An attenuator was used to obtain the signal of water protons. The longitudinal relaxation rate (*R*1, 1/ms) and transverse relaxation rate (*R*2, 1/ms) were calculated from *T*1 and *T*2, where *R*1 = 1/*T*1, *R*2 = 1/*T*2. The longitudinal relaxivity (*r*1, 10^−3^ M^−1^·s^−1^) and transverse relaxivity (*r*2, 10^−3^ M^−1^·s^−1^) were calculated by a linear regression of *R*1, *R*2 versus different Gd concentrations.

The *T*1-weight images were obtained in a 1.5 T MRI system with a repetition time (TR) of 200 ms, an echo time (TE) of 50 ms, and a 128 × 168 image acquisition matrix. The synthesized PEG contrast agents were dissolved in distilled water (concentration: 0.05 mM–0.5 mM). The luminance was calculated from the pixel of the histogram using Photoshop software (Adobe Co. Ltd., California, USA).

### 2.5. Terminal Mobility

The terminal mobility of PEG-Gds having different structures was determined using a 300 MHz (7.1 T) NMR spectrometer at various temperatures. Each synthesized PEG-Gd was dissolved in deuterium oxide to a concentration of 15 wt/wt.%. For each temperature (25, 37, and 60°C), the solution was equilibrated for 30 min before acquiring the data. The specificity of the solvent was also evaluated using deuterated solvents of various polarities, dimethyl sulfoxide-d_6_ (DMSO-d_6_), methanol-d_4_ (CD_3_OD), and chloroform-d (CDCl_3_).

## 3. Results and Discussion

### 3.1. Synthesis of PEG Contrast Agents

To clarify the relation between the polymer structure and the contract efficiency of the contrast agents, linear PEG (Mn = 10000), dendron PEG (Mn = 40000), four-arm PEG (Mn = 15000), eight-arm PEG (Mn = 15000), and commercially available contrast agent shown in Scheme 1(a) were used. The terminal amino groups of PEG were reacted with DOTA-NHS-ester to obtain PEG-DOTA (Scheme 1(b) Step 1). The terminal DOTA ratio was confirmed by NMR measurements: 0.73 (DOTA/PEG(mol/mol)) for linear PEG-DOTA, 0.65 (DOTA/PEG(mol/mol)) for dendron PEG-DOTA, and 1.8 (DOTA/PEG(mol/mol)) for four-arm PEG-DOTA. Additionally, different DOTA introduction ratios from 1.78 to 3.95 (DOTA/PEG(mol/mol)) of the eight-arm PEG-DOTAs were synthesized (data not shown). The obtained PEG-DOTAs were subsequently reacted with GdCl_3_ overnight to obtain PEG-Gd (Scheme 1(b) Step 2), and the Gd introduction ratio was confirmed by ICP-MS.  Table 1 shows the summary of the synthesized PEG-Gd with different structures. The Gd introduction ratios were as follows: 0.70 (Gd/PEG (mol/mol)) for linear PEG-Gd, 0.69 (Gd/PEG (mol/mol)) for dendron PEG-Gd, 1.97 (Gd/PEG (mol/mol)) for the four-arm PEG-Gd, and 1.97 to 4.07 (Gd/PEG (mol/mol)) for the eight-arm PEG-Gds. The Gd introduction ratios were similar to the DOTA ratio, which was confirmed by that DOTA chelate formed a complex with Gd; subsequently, the PEG-Gd contrast agents were synthesized successfully. Moreover, the impurities were not detected on the NMR spectrum. All the synthesized PEG-Gds could dissolve in water. The PEG-Gd having a Gd introduction ratio of 5 (Gd/PEG (mol/mol)) was also synthesized, but it could not be dissolved in water (data not shown).

Table 1 also shows the zeta potential and hydrodynamic radius of the synthesized PEG-Gd and commercially available contrast agents, Magnescope (Guerbet Co. Ltd., Villepinte, France) and ProHance (Bracco Eisai Co. Ltd., Tokyo, Japan). The zeta potential of Magnescope was slightly negative, whereas that of ProHance was slightly positive. The zeta potential of eight-arm (1.8) and four-arm (2.0) was slightly positive because of the remaining terminal amino group. The hydrodynamic radii are as follows: 4 nm for linear PEG-Gd and four-arm PEG-Gd, 11 nm for dendron PEG-Gd, and approximately 3 nm for the eight-arm PEG-Gds. This implies that the hydrodynamic radius depended on the molecular weight. The commercially available products were approximately 1 nm. Furthermore, large particles due to aggregation or undissolved PEG-Gd were not observed in all solutions (concentration: 1 wt/wt.%), suggesting no aggregates.

### 3.2. Relaxivity and *T*_1_-Weighted Image of Synthesized PEG Contrast Agents

**Figure 1:**
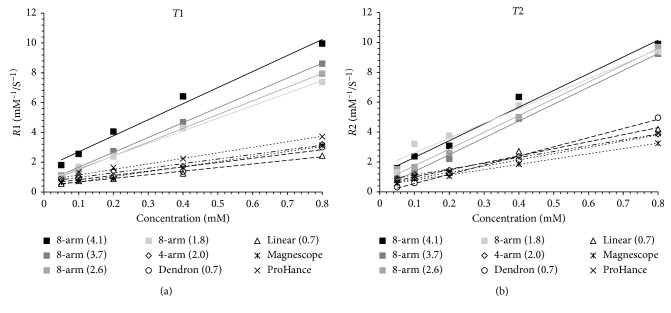
Relaxivity measurements of synthesized and commercially available contrast agents.

The relaxivities *r*1 and *r*2 of the synthesized PEG-Gd contrast agents and commercially available contrast agents were calculated from the regression analysis of *R*1 and *R*2 relative to the Gd concentration, as shown as Figure 1. The gradient of the eight-arm PEG-Gd contrast agents was more oblique than that of the other contrast agents. The Gd chelate used in this study reduces the *T*1 and *T*2 values of tissue water; therefore, they cause positive enhancement in *T*1-weighted images and negative enhancement in *T*2-weighted images. The relaxivities of the synthesized PEG-Gd contrast agents and commercially available contrast agents are summarized in Table 2. The relaxivity *r*1 of the branched PEG-Gds were higher than that of linear PEG-Gd, dendron PEG-Gd, and commercially available contrast agents. Moreover, the relaxivity was increased with the number of PEG branches. The eight-arm PEG-Gds showed the highest *r*1 of 10.77 mM^−1^·s^−1^, which was approximately 3 times higher than the relaxivity of commercially available contrast agents. Although the terminal Gd conjugation is different, it did not affect *r*1. Although dendron PEG-Gd is branch structured, its *r*1 value was low because the Gd chelate did not react at the branched terminal group. The *r*2 of the negative enhancement in *T*2-weighted images also shows a higher value at the branched structure, and the *r*2/*r*1 values of eight-arm PEG-Gds were 1.1 to 1.2, which were the most similar to the values of the commercially available positive contrast agent.

**Figure 2:**
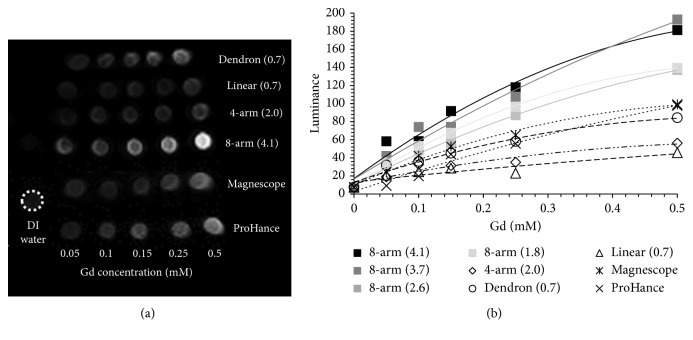
(a) MR imaging of *T*_1_-weighted MRI measurements and (b) luminance calculated from the pixel of the histogram, of synthesized and commercially available contrast agents in water at 1.5 T at the concentrations of 0.05, 0.1, 0.15, 0.25, and 0.5 mM.

Figure 2 shows the *T*1-weighted MRI measurements of a synthesized PEG-Gd contrast agent and a commercially available contrast agent. In Figure 2(a), the eight-arm PEG-Gd shows the brightest contrast. The luminance was calculated and summarized in Figure 2(b). The luminance at Gd concentration 0 mM, using DI water as blank, is 7.5. The eight-arm (4.1) and eight-arm (3.7) show the highest luminance of 180, which is approximately 2 times higher than the luminance of commercially available contrast agents. The luminance of eight-arm (2.6) and eight-arm (1.8) is 140. The luminance of linear (0.7), dendron (0.7), and four-arm (2.0) is 40, 80, and 60, respectively, which is lower than the commercially available contrast agents. Figure 2 shows the luminance of dendron PEG-Gd is brighter than that of 4-arm PEG-Gd. In contrast, *r*1 of 4-arm PEG-Gd (*r*1 = 3.09) is slightly higher than dendron PEG-Gd (*r*1 = 2.87) (Figure 1). This slight *r*1 difference between 4-arm PEG-Gd and dendron PEG-Gd may not precisely reflect in MRI (1.5 T) because of the unbalanced magnetic field of MRI. However, 8-arm PEG-Gd certainly had clear differences compared to others.

### 3.3. Terminal Mobility of PEG Contrast Agents

**Figure 3:**
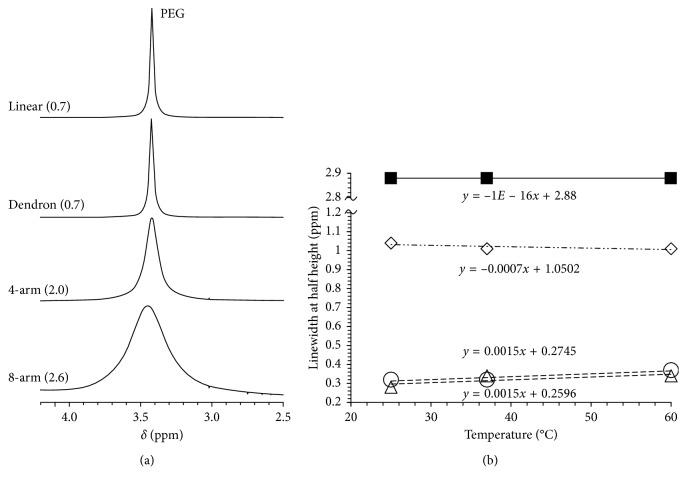
(a) ^1^H NMR measurements of the synthesized PEG-Gd at room temperature. (b) Evaluation of mobility of PEG-Gd (■ eight-arm (2.6), ◊ four-arm (2.0), ○ dendron (0.7), and △ linear (0.7)) in D_2_O at various temperatures.

We used ^1^H NMR measurements to study the mobility of the synthesized PEG-Gd contrast agents (15 wt/wt.%) in D_2_O. Their ^1^H NMR spectra are shown in Figure 3, and the position of the water peak at 4.54 ppm was used as the internal reference. The PEG peak at 3.46 ppm was used to calculate the linewidth at half height of each PEG-Gd.

It has been demonstrated that when the molecular mobility is low, the relaxation time became shorter and the peak became broader. As shown in Figure 3(a), the peak became broader with the number of PEG branches increased. The linewidth at half height of each PEG-Gd at different temperatures is shown at Figure 3(b). The eight-arm (2.6) showed the broadest linewidth at half height, which was 10 times broader than the linewidth at half height of linear (0.7) and dendron (0.7) and did not change with temperature change. Meanwhile, the linewidth at half height of four-arm (2.0) was also broader than that of linear (0.7) and dendron (0.7) but was much narrower than eight-arm (2.6). Linear (0.7) and dendron (0.7) showed the narrowest linewidth at half height, and they became broader slightly when the temperature increased. These results suggested that the mobility of PEG having a branched structure was stable in water, even when the temperature changed.  Table 1 shows that no large particles appeared in the eight-arm PEG-Gd solution, suggesting that the stable mobility was not derived from the aggregates. Because the arm is short in a single molecular chain, the mobility is low and the molecule is considered as stable.

It has been reported that the relaxivity is affected by water accessibility and the molecular mobility [25, 29, 30]. The increase in relaxivity observed upon addition of the PEG chain is very modest. This is due to the decrease in the number of coordinated water molecules and the effect of rapid internal motions of the PEG chain on rotational correlation lifetime [25]. Toth et al. reported that the rotational correlation time of the Gd(DTPA-BA)-PEG polymer is restricted to its monomer chelate unit rotated around a single axis, owing to the internal flexibility of the PEG moieties. Consequently, the relaxivities of this polymer are slightly greater than that of Gd(DTPA-BMA) [29]. In our research, eight-arm PEG-Gd showed higher relaxivity; because of the shorter PEG chain in one arm (Mn = 15000/8 = 1875 Da), its Gd chelate unit restricted to rotate around a single axis. In contrast, the linear PEG-Gd showed slightly lower relaxivity, which may be because of the higher flexibilities of the long chain of PEG (Mn = 10000 Da) and free rotation of Gd chelate.

**Figure 4:**
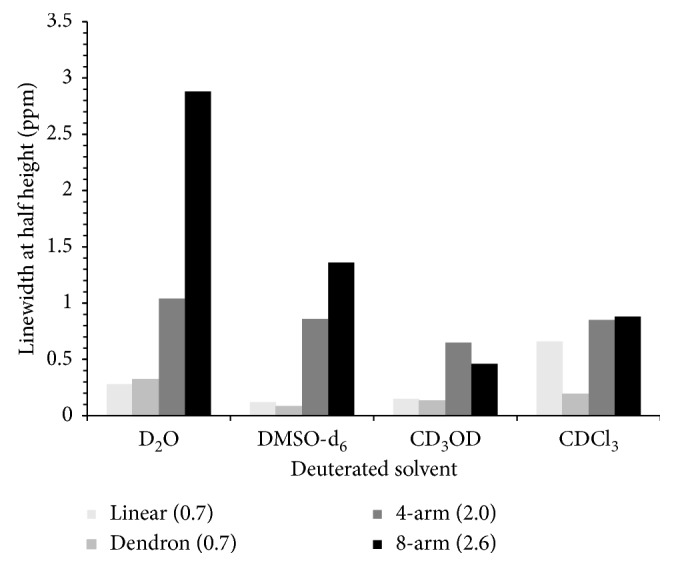
Evaluation of mobility of the contrast agent in different solvents at room temperature.

The molecule mobility influenced by the solvent was also evaluated using deuterated solvents of various polarities, DMSO-d_6_, CD_3_OD, and CDCl_3_. As mentioned before, when the molecular mobility became lower, the relaxation time of water became shorter, and the peak of NMR became broader.  Figure 4 shows the evaluation of the mobility of PEG-Gd contrast agents in different solvents at room temperature. Four types of deuterated solvents with various solvent polarities were used. D_2_O and CD_3_OD are protic solvents, DMSO-d_6_ is an aprotic solvent, and CDCl_3_ is a nonpolar solvent. The eight-arm (2.6) showed a broader linewidth at half height in D_2_O than in CDCl_3_, and linear (0.7), dendron (0.7), and four-arm (2.0) showed no significant difference with the deuterated solvent changed.  Figure 4 is the first direct observation of strongly restricted mobility of eight-arm PEG-Gd in water. These results show the molecular mobility of eight-arm PEG-Gd was specifically influenced by water, leading to the shorter relaxation time, higher relaxivity, and higher contrast efficiency.

## 4. Conclusions

Water-soluble PEG-Gd contrast agents were synthesized in a two-step reaction. The relaxivity and luminance of branch-structured PEG-Gds were higher than those of linear-structured PEG-Gd. Although the terminal Gd conjugation is different, it did not affect the longitudinal relaxivity and luminance in the eight-arm PEG-Gd. The eight-arm PEG-Gd shows the highest contrast efficiency and is even higher than that of commercially available contrast agents. The linewidth at half height measured by NMR became broad when the number of PEG branches increased, implying that the mobility of branched PEG-Gd is slower than that of linear PEG-Gd. This phenomenon appeared specifically in water. We conclude that the stable branched structure enabled the improvement in the relaxivity and contrast luminance.

## Figures and Tables

**Table 1:** Summary of the synthesized PEG-Gd with different structures.

Product	MW (data sheet)	Gd introduction ratio (Gd/PEG (mol/mol)) (ICP-MS)	Zeta potential (mV)	Hydrodynamic radius (nm)	Yield (%)
8-arm (4.1)	15000	4.07	−0.22	2.90	90.7
8-arm (3.7)	15000	3.70	−0.02	3.05	74.9
8-arm (2.6)	15000	2.64	−0.02	2.24	94.8
8-arm (1.8)	15000	1.79	0.08	2.70	97.2
4-arm (2.0)	15000	1.97	0.04	4.04	42.6
Dendron (0.7)	40000	0.69	−0.08	11.72	99.2
Linear (0.7)	10000	0.70	−1.73	4.00	54.2
Magnescope	559	—	−0.11	0.99	—
ProHance	559	—	0.18	1.07	—

**Table 2:** Relaxivity of the synthesized PEG-Gd with different structures compared to commercial contrast agents.

Product	*r*1	*r*2	*r*2/*r*1
8-arm (4.1)	10.77	11.20	1.04
8-arm (3.7)	9.97	11.27	1.13
8-arm (2.6)	9.22	11.22	1.22
8-arm (1.8)	8.42	9.91	1.18
4-arm (2.0)	3.09	3.92	1.27
Dendron (0.7)	2.87	6.14	2.14
Linear (0.7)	2.42	4.72	1.95
Magnescope	3.38	3.54	1.05
ProHance	3.71	4.19	1.13

## Data Availability

The data used to support the findings of this study are included within the article.
